# Novel Baseline Facial Muscle Database Using Statistical Shape Modeling and In Silico Trials toward Decision Support for Facial Rehabilitation

**DOI:** 10.3390/bioengineering10060737

**Published:** 2023-06-19

**Authors:** Vi-Do Tran, Tan-Nhu Nguyen, Abbass Ballit, Tien-Tuan Dao

**Affiliations:** 1 Faculty of Electrical and Electronics Engineering, Ho Chi Minh City University of Technology and Education, Thu Duc City 71300, Ho Chi Minh City, Vietnam; dotv@hcmute.edu.vn; 2 School of Engineering, Eastern International University, Thu Dau Mot City 75100, Binh Duong Province, Vietnam; nhu.nguyentan@eiu.edu.vn; 3 Univ. Lille, CNRS, Centrale Lille, UMR 9013-LaMcube-Laboratoire de Mécanique, Multiphysique, Multiéchelle, F-59000 Lille, France; abbass.ballit@centralelille.fr

**Keywords:** facial muscle baseline, statistical shape modeling, in silico trials, facial rehabilitation, facial muscle quantification, clinical decision-support system

## Abstract

**Backgrounds and Objective**: Facial palsy is a complex pathophysiological condition affecting the personal and professional lives of the involved patients. Sudden muscle weakness or paralysis needs to be rehabilitated to recover a symmetric and expressive face. Computer-aided decision support systems for facial rehabilitation have been developed. However, there is a lack of facial muscle baseline data to evaluate the patient states and guide as well as optimize the rehabilitation strategy. In this present study, we aimed to develop a novel baseline facial muscle database (static and dynamic behaviors) using the coupling between statistical shape modeling and in-silico trial approaches. **Methods**: 10,000 virtual subjects (5000 males and 5000 females) were generated from a statistical shape modeling (SSM) head model. Skull and muscle networks were defined so that they statistically fit with the head shapes. Two standard mimics: smiling and kissing were generated. The muscle strains of the lengths in neutral and mimic positions were computed and recorded thanks to the muscle insertion and attachment points on the animated head and skull meshes. For validation, five head and skull meshes were reconstructed from the five computed tomography (CT) image sets. Skull and muscle networks were then predicted from the reconstructed head meshes. The predicted skull meshes were compared with the reconstructed skull meshes based on the mesh-to-mesh distance metrics. The predicted muscle lengths were also compared with those manually defined on the reconstructed head and skull meshes. Moreover, the computed muscle lengths and strains were compared with those in our previous studies and the literature. **Results**: The skull prediction’s median deviations from the CT-based models were 2.2236 mm, 2.1371 mm, and 2.1277 mm for the skull shape, skull mesh, and muscle attachment point regions, respectively. The median deviation of the muscle lengths was 4.8940 mm. The computed muscle strains were compatible with the reported values in our previous Kinect-based method and the literature. **Conclusions**: The development of our novel facial muscle database opens new avenues to accurately evaluate the facial muscle states of facial palsy patients. Based on the evaluated results, specific types of facial mimic rehabilitation exercises can also be selected optimally to train the target muscles. In perspective, the database of the computed muscle lengths and strains will be integrated into our available clinical decision support system for automatically detecting malfunctioning muscles and proposing patient-specific rehabilitation serious games.

## 1. Introduction

Patients with facial palsy have difficulties in their daily activities for interpersonal communicating and expressing emotions, so facial mimic rehabilitation can enhance the life quality of the involved patients [1,2]. Facial mimics have resulted from muscle activations on skin layers [3,4,5]. The muscle activations were controlled by motor nerves [6]. Some causes (e.g., strokes and facial transplants) could make these nerves dysfunctioned so that they cannot activate their target muscles [7]. Consequently, the patients could not naturally and symmetrically perform some mimics (e.g., smiling and kissing) controlled by these malfunctioned muscles [8] or have unwanted facial movements in neutral or dynamic mimics [9]. The recovery procedures for these muscles were complex and needed long-term treatments [10,11,12,13,14,15]. Facial rehabilitation exercises can enhance recovery speed and treatment performances [16]. These exercises include repetitive and simple facial movements dedicated to specific muscles [8]. Consequently, dysfunctioned facial muscles must be first analyzed and diagnosed before selecting suitable types of exercises.

Regarding facial paralysis analysis, clinical and non-clinical facial paralysis grading methods have been employed [17]. Clinical facial paralysis grading, which was mainly based on the expertise of clinicians, was very subjective and varied among clinicians [17,18,19]. However, the non-clinical facial paralysis grading, which was mainly based on computer-aided processes, was objective and not dependent on clinicians [17]. In the literature, most studies tried to analyze facial mimics by evaluating their external geometrical information [20]. This information could be the symmetries between the left and right faces on 2D images or 3D meshes [21,22,23,24]. Moreover, symmetrical movements of 2D and/or 3D face features could also be employed [25,26,27,28,29,30,31,32,33]. Some studies also tried to detect and analyze action units (AUs) of the Facial Action Coding Systems (FACS) [34] through 2D images or 3D point clouds [20,35]. However, facial mimics are deformation results of muscle contractions on skin layers [3,4,5], so muscle behaviors should be directly analyzed instead of these geometrical appearances. In our previous study, we first proposed the concept of using muscle strains for facial paralysis grading [36]. A muscle network could be statistically predicted based on the target head shape and a statistics-based predicted skull mesh [37]. During the real-time head animation, we could fast compute muscle strains according to the vertex movements on the head and skull meshes [36]. However, we lacked standard values of muscle strains for diagnosing these muscles. Moreover, we only report muscle lengths and strains of only five subjects (three healthy subjects and two patients), so these values could not be represented for large populations during the muscle diagnoses [36].

Measuring standard muscle parameters was relatively challenging. In experimental studies, skeletal muscle measurements of faces could be conducted on cadavers, but the number of subjects was relatively small (from 1 to 20) [38,39,40]. These processing procedures needed much clinical expertise. In in-silico studies, facial muscles could be reconstructed through magnetic resonance imaging (MRI) data, but segmenting soft tissues in MRI images is time-consuming and need much clinical expertise [4,5]. Consequently, we could not reconstruct all facial muscles for a large number of subjects using this MRI-based method. Using computed tomography (CT) imaging data, although we can reconstruct both head and skull meshes, the soft tissues are lacking [37]. Moreover, most cadaver, MRI, or CT datasets were collected from dead subjects, so the measurement cannot be conducted in different mimics, especially in a dynamic manner [4,5,37,38,39,40]. In silico trials have been popularly employed for fastening novel clinical treatments and experiments. In in-silico trials, novel medical treatments or experiments were tested on the personalized virtual human models for fast collecting responses in simulation environments. These responses were employed for optimizing the treatments before being implemented on the real human. Consequently, in-silico trials help reduce clinical costs and deal with the lack of experimental data [41,42].

Recently, the statistical shape modeling (SSM) method has been popularly employed for modeling the human head and/or face geometries and mimics (i.e., FLAME head model [43], Basel face model [44], and other 3D morphable models [45]). These models were trained on large datasets of human faces both in static and dynamic mimics reconstructed from accurate depth sensors, so they can be used for representing the shapes and mimics of large populations [46]. These morphable face/head models were popularly employed for predicting face head meshes on mono images of large face variations [47]. Although these SSM models could generate large variations in head shapes and facial mimics, they still lack internal structures (i.e., skulls and muscle networks). In our previous study, we could predict internal structures including the skull and muscle network with acceptable accuracy for both patient and healthy subjects [36,37], but this prediction method has not been applied to those SSM head models.

Consequently, in this study, we aimed to apply our previous biomechanical head modeling methods [36,37] to the FLAME (Faces Learned with an Articulated Model and Expressions) head model [43] for computing standard values of muscle lengths and strains in static and dynamic facial mimics. In particular, the FLAME head model can generate 3D geometrical head models that are the 3D triangulated surface meshes of the head shapes. The vertices positions of the head meshes are controlled by the parameters of subject identity, head transforms, and facial mimics. The skull meshes were predicted based on the head meshes so that their shapes statistically fit with the head shapes. Muscle networks were defined as the action lines connected from the muscle attachment points on the skull to the muscle insertion point on the head. The muscle lengths and strains were finally computed in standard static and dynamics mimics because they are important to muscle-based facial paralysis diagnosing. These values will be applied in our clinical decision-support system for facial mimic rehabilitation. In the system, we can compare the computed static and dynamic muscle strains with the reported baseline values for automatically detecting malfunctioned facial muscles. In this study, the baseline facial muscle database is the database of the static and dynamic lengths and strains of the facial muscles in the kissing and smiling mimics. Moreover, suitable types of facial rehabilitation games will be proposed to train the detected muscles. The muscle behaviors will also be scored based on these standard values during playing games for evaluating the recovery progresses.

In the following sections, we will first describe the methods of head shape and mimic generations, skull prediction, and muscle network definition. The steps of CT-based validation will also be presented in the Section 2. The baseline values of muscle lengths and strains in neutral and other mimics according to their accuracies will also be reported in the Section 3. The contributions and drawbacks of this study will finally be discussed in the Section 4 and Section 5.

## 2. Materials and Methods

### 2.1. Overall Processing Workflow

**Figure 1:**
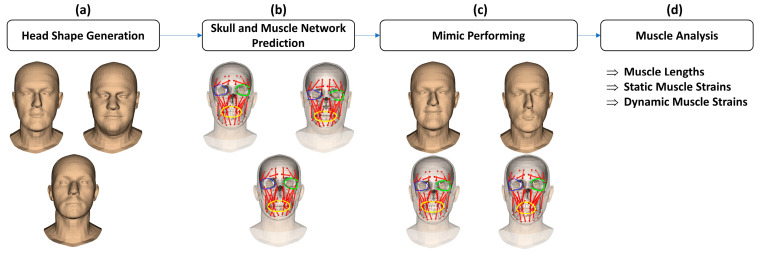
The overall processing procedure for analyzing the facial muscle’s behaviors for both static and dynamic mimic positions: (**a**) the shape variations were defined thanks to the statistical shape model of the head; (**b**) skulls and muscle networks were defined based on the head shapes; (**c**) the static and dynamic mimics were performed by the virtual subjects and drove the skull and muscle network’s structures; (**d**) muscle lengths, static and dynamic muscle strains were computed based on the current muscle movements. Note that the red, green, blue, and yellow colors are represented the linear muscles, left orbicularis muscle, right orbicularis muscle, and oris muscle.

The overall processing procedure is described in Figure 1. In particular, the processing steps include (a) head shape generation, (b) skull and muscle network prediction, (c) mimic performing, and (d) muscle analysis.

(a) Regarding head shape generation, we use the SSM head model, Faces Learned with an Articulated Model and Expressions (FLAME) [43], for generating variations in virtual subjects by controlling the FLAME shape parameters. The other parameter sets including translations, rotations, poses, and expressions were kept all to zeros to be on the neutral mimic positions. The details of this step are explained in Section 2.2.
(b) Regarding the skull and muscle network prediction, based on the head shape of each virtual subject, a skull mesh was predicted thanks to our developed SSM-based head-to-skull prediction method [43]. Moreover, a muscle network including linear and circle muscles was defined as action lines connected from muscle attachment points on the skull mesh to the muscle insertion points on the head mesh. The insertion and attachment points were positioned based on their vertex indices on the head and skull meshes. This processing step is clearly explained in Section 2.3.
(c) Regarding mimic performing, we controlled the expression and pose parameters of the FLAME model to perform smiling and kissing mimics on each virtual subject. In the static mimics, we set the max values on the smiling and kissing control parameters. In the dynamic mimics, we set these parameters from zero to their max values with the step size of 1/200 of these max values. The details are explained in Section 2.4.
(d) Regarding muscle analysis, because the mesh structures of the head and skull meshes do not change during the non-rigid animations, muscle insertion, and attachment points were automatically updated according to the motions of head and skull vertices. Consequently, muscle lengths could also be computed according to the updated insertion and attachment points. Muscle strains were computed as relative differences between the muscle lengths in the current mimics and those in the neutral mimics. In this study, muscle strains of both static and dynamic mimics were computed and reported. The details are presented in Section 2.4.

For validation, we tested the methods on the 5 head and skull meshes reconstructed from the CT image sets from the New Mexico Decedent Image Database [48]. The predicted skull meshes were compared with the reconstructed skull meshes based on the mesh-to-mesh distance metric. Moreover, the predicted muscle networks were also compared with the pre-defined muscle networks on the CT-based head and skull meshes based on the point-to-point distance metric. Last but not least, the computed muscle lengths were also compared with the reported muscle lengths in the literature. Details of the validation are explained in Section 2.5.

### 2.2. Subject Identity and Mimic Generation

Faces Learned with an Articulated Model and Expressions (FLAME) head model was one of the most popular 3DMM (3D Morphable Models) for the human head [43]. The FLAME model employed the non-animated head mesh of the SMPL (Skinned Multi-Person Linear) model [49]. The head mesh vertices were formed as Equations (1) and (2).

(1) $$M\left(\vec{\beta },\;\vec{\theta },\vec{\psi }\right)=W\left(T_{P}\left(\vec{\beta },\;\vec{\theta },\vec{\psi }\right),J\left(\vec{\beta }\right),\;\vec{\theta },𝒲\right)$$

(2) $$T_{P}\left(\vec{\beta },\;\vec{\theta },\vec{\psi }\right)=\bar{T}+B_{S}\left(\vec{\beta },𝒮\right)+B_{P}\left(\vec{\theta },𝒫\right)+B_{E}\left(\vec{\psi },\;ℰ\right)$$

In which, $J\left(\vec{\beta }\right)$ are the joint locations of the rotation parts on the head mesh (mouth mesh, left eye mesh, and right eye mesh), and they were computed from the head vertices. 𝒲 are blend weights for linearly smoothing the skin vertices during rotating around the joints $J\left(\vec{\beta }\right)$. $\bar{T}$ is the mean head vertices computed from the training dataset. $B_{S}\left(\vec{\beta },𝒮\right)$ is a shape blend shape function with the shape parameters, $\vec{\beta }$, and orthonormal shape basis, 𝒮. The 𝒮 was trained from the training dataset using the principal component analysis (PCA) [50]. $B_{P}\left(\vec{\theta },𝒫\right)$ is a pose blend shape function with the pose parameters, $\vec{\theta }$, and the vertex offset from the rest pose, $𝒫.\;B_{E}\left(\vec{\psi },\;ℰ\right)$ is the expression blend shape function with the expression parameters, $\vec{\psi }$, and the orthonormal expression basis, ℰ. The ℰ was trained from the dataset using the PCA.

The training dataset of the FLAME head model includes 3800 scanned heads from the European CAESAR body scan database [51]. This dataset was scanned by 6 infrared time-of-flight sensors put around the target body with the circumferential accuracy <±5 mm. A template head mesh was deformed to the head shapes of this dataset to form the dataset for training the shape blend shape function. Moreover, the face expression model was also trained on the D3DFACS dataset [52], which contained 3D point cloud sequences of the face in time series with various standard facial expressions (facial action units (AUs)) defined in the facial action coding system (FACS) [53]. The template head meshes with pre-defined shape parameter sets was deformed to each facial to form the dataset for training the pose and expression blend shape functions. The FLAME head model was trained separately for males, females, and generic datasets.

**Figure 2:**
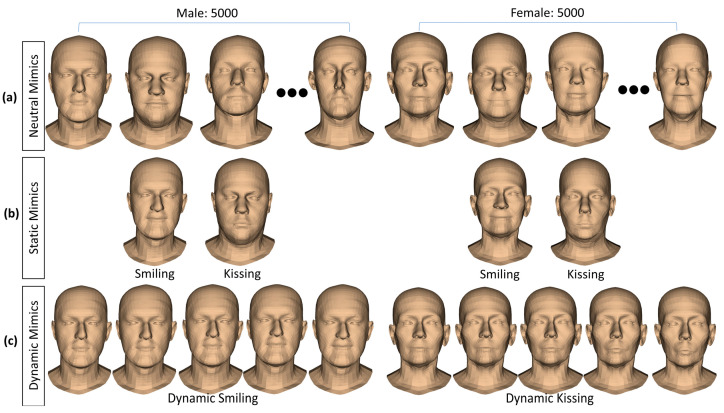
Subject identities and mimics generations from the statistical shape model of the head: (**a**) shape variations in neutral mimics of 5000 male subjects and 5000 female subjects; (**b**) static mimics performed by the virtual subjects; (**c**) dynamical mimics in time series.

Consequently, using the FLAME head model, we can re-generate the head mesh with various shapes and realistic facial expressions. we re-generated 10,000 virtual subjects with 10,000 shape parameter sets (5000 for males and 5000 for females). Figure 2a shows examples of head shape variations in neutral mimics of 10,000 virtual male and female subjects. To vary the subject identity of the FLAME head mesh, we tried to set the FLAME’s pose and mimic parameters to zeros so that the head and jaw regions were in the standard position, and the faces were in the neutral mimic. All shape parameters of the FLAME were randomly valued from their minimal to maximal (from −2.0 to 2.0). This selection strategy will guarantee that the regenerated head meshes will cover most variations in the head shapes on the FLAME’s training dataset. Additionally, two separate male and female models of the FLAME were employed for re-generating male and female virtual subjects.

For each virtual subject, we changed the expression parameters of the FLAME to their maximum values for creating static smiling and kissing mimics, as shown in Figure 2b. Moreover, dynamic mimics of each type of face movement (smiling and kissing) were also created by setting the appropriate expression parameters from zeros to their max values, as described in Figure 2c. 

### 2.3. Skull and Muscle Network Generation

**Figure 3:**
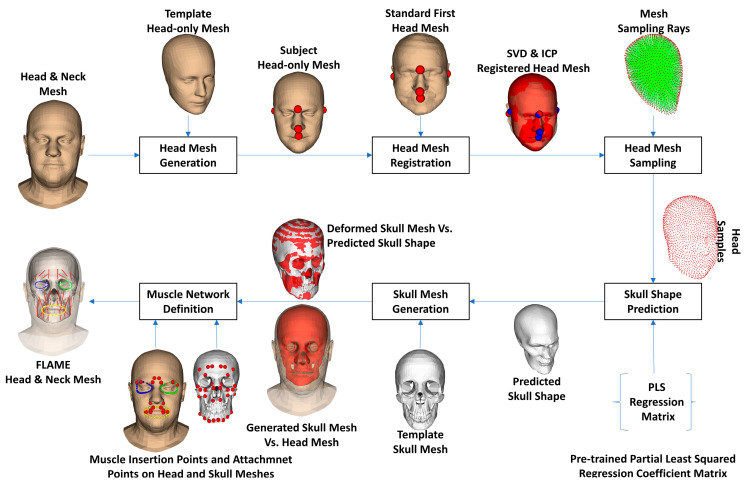
Skull and muscle network generation procedure. Note that the color dots in this figure represent the muscle insertion/attachment points and feature points for rigid transformation.

Even though the FLAME model can be used to generate shapes and mimics for large populations, they still lack the internal structures for analyzing muscle behaviors in each mimic. The head-to-skull prediction method was developed in our previous study, but we just applied it to the CT and Kinect-based head meshes [37]. In this study, we tried to apply the method to the head meshes generated from the FLAME model. Figure 3 shows the processing procedure of the FLAME-based head-to-skull prediction. In particular, given a neutral head-neck mesh generated by the FLAME model, we first generated the head-only mesh by replacing the vertices of the template head-only mesh with the appropriate vertices on the FLAME-based head-neck mesh. The head-only mesh was then registered to the standard head mesh based on the manually selected landmarks. The singular value decomposition (SVD) [54] and iterative closest point (ICP) registration methods [55] were employed for minimizing the manual landmark selections. The registered head mesh was sampled using the sampling rays, which were pre-defined during the head-to-skull training processes, to result in the head samples. A skull shape was predicted from the head samples using the partial least squared regression coefficients [56], which were trained in our previous study [37]. A template skull shape was finally deformed so that its shape optimally fitted with the generated skull shape. 

**Figure 4:**
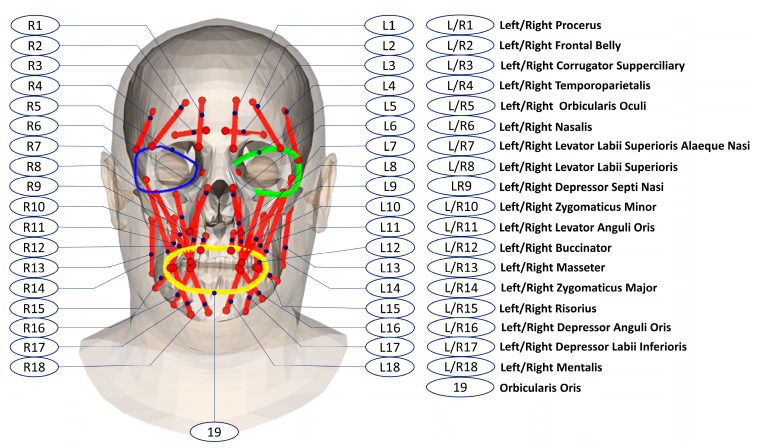
Muscle network definitions on the FLAME head mesh and the skull mesh.

As shown in Figure 4, a muscle network was defined as action lines connecting from attachment points on the skull mesh to the attachment points on the head (or skull) mesh. We defined facial muscle types based on the face’s anatomical structure [57]. The defined linear muscles included the Left/Right Procerus (L/RP), Left/Right Frontal Belly (L/RFB), Left/Right Corrugator Supperciliary (L/RCS), Left/Right Temporoparietalis (L/RT), Left/Right Nasalis (L/RN), Left/Right Depressor Septi Nasi (L/RDSN), Left/Right Zygomaticus Minor (L/RZm), Left/Right Zygomaticus Major (L/RZM), Left/Right Risorius (L/RR), Left/Right Depressor Anguli Oris (L/RDAO), Left/Right Mentalis (L/RM), Left/Right Levator Labii Superioris (L/RLLS), Left/Right Levator Labii Superioris Alaeque Nasi (L/RLLSA), Left/Right Levator Anguli Oris (L/RLAO), Left/Right Depressor Labii Inferioris (L/RDLI), Left/Right Buccinator (L/RB), and Left/Right Masseter (L/RMa). We also defined circle muscles including Left/Right Orbicularis Oculi and Orbicularis Oris. The muscle insertion/attachment points were positioned by the vertex indices on the head and skull meshes. During the animation of the head and skull meshes, the mesh vertex indices were not changed, so the muscle lengths and perimeters could be updated according to their insertion and attachment positions on the animated head and skull meshes. 

### 2.4. Muscle-Based Analyses

In this study, we analyze the muscle behaviors in neutral, static mimics, and dynamic mimics. In neutral mimics, the lengths of linear muscles were computed as distances between their attachment points and insertion points. The mean and standard deviation lengths of each muscle throughout all male and female subjects were computed and reported. In static mimics, for each subject, during performing smiling and kissing, the length of each muscle was computed for each mimic. Relative differences between their lengths in the current mimics and those in the neutral mimics were computed as their muscle strains. The mean and standard deviation strains of each muscle throughout all male and female subjects were computed and reported for each mimic. In dynamic mimics, for each type of mimic (smiling or kissing), the values of expression parameters were increased from zeros to their max values with the step size of 1/200 of the max values and set to the FLAME model to generate the mimics. Throughout all male and female subjects, the mean and standard deviation strains of each muscle were computed and reported for each small mimic.

### 2.5. Validation

**Figure 5:**
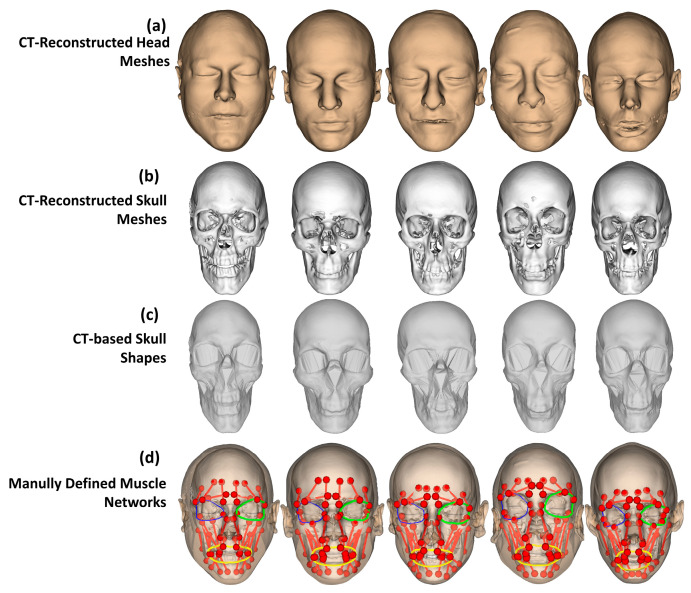
Validation data reconstructed and defined from CT images: (**a**) reconstructed head meshes, (**b**) reconstructed skull meshes, (**c**) generated skull shapes from the CT-based skull meshes, and (**d**) muscle network manually defined based on the face anatomical structures and reconstructed head and skull meshes. The colored lines represent muscle action lines in different muscle types: linear, left oculi, right oculi, and oris for red, green, blue, and yellow colors.

To evaluate the accuracies of head-to-skull prediction and muscle network definition. We collected five CT image sets from the New Mexico Decedent Image Database [48] (males: 3, females: 2, ages (Mean ± SD): 31.2 ± 6.5 years old). The CT image sets were reconstructed using the 3D Slicer software [58]. The head meshes were reconstructed by first segmenting both skins and bones in the CT images and then meshing based on the marching cube methods. Internal structures and neck regions were removed from the reconstructed meshes, as shown in Figure 5a. The skull meshes were reconstructed by first segmenting the bone tissue in the CT images and then meshing the segmented regions. The cervical spines were also removed from the reconstructed skull meshes, as shown in Figure 5b. Moreover, we also generated skull shapes from the CT-reconstructed skull meshes for shape validation, as shown in Figure 5c. The details of skull shape generation from skull meshes were described in our previous study [37]. The muscle networks were also manually defined by selecting their attachment points and insertion points on the reconstructed skull meshes and head meshes, as shown in Figure 5d, based on the face anatomy [57]. We applied our previous head-to-skull prediction method [37] and muscle network definition method [36] for predicting the skull meshes and muscle networks for the five CT-reconstructed head meshes. The skull shapes of the predicted skull meshes were also generated for evaluation. Distances between the predicted skull shapes and the CT-based skull shapes and between the predicted skull meshes and the CT-reconstructed skull meshes were computed based on the mesh-to-mesh distance metric. The computed mesh-to-mesh distances were also evaluated in muscle attachment/insertion point regions on the skull meshes. Additionally, we also compared the muscle lengths of the manually defined muscle networks and the predicted muscle networks for evaluating the accuracy of the muscle network’s prediction using the point-to-point distance metric. The predicted muscle lengths in neutral mimics and linear muscle strains in smiling and kissing mimics were also compared with those reported in the literature [5,38,39,40] and our previous study [36].

### 2.6. Used Technologies

The head mesh generation, skull prediction, and muscle parameter computing was programmed in Visual Studio C++ 2019 in the hardware configuration of HP Zbook 17G5 Intel(R) Xeon(R) E-2176M CPU @ 2.70GHz 2.71 GHz, 32.0 GB RAM, 64 bits Microsoft Windows 11 Pro for Workstations. Mesh processing was supported by LibIGL [59] and VCG and MeshLab [60]. Point cloud processing was supported by PCL C++ [61] libraries. The mesh rendering was supported by VTK [62]. The linear matrix operation was supported by Eigen [63]. The FLAME head model execution was executed on Tensorflow C++ API [64].

## 3. Results

### 3.1. Validation Deviations in Comparison with CT-Reconstructed Data

**Figure 6:**
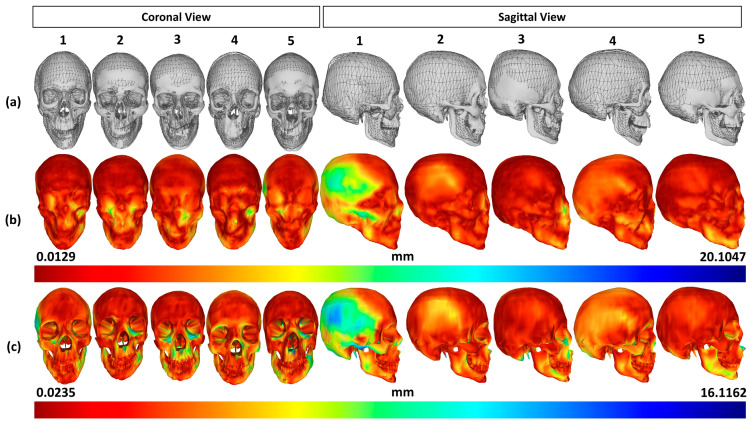
Validation results of the predicted skull mesh: (**a**) predicted skull meshes vs. reconstructed skull meshes, (**b**) error distribution in distance color maps between the predicted skull shapes and the reconstructed skull shapes, and (**c**) error distribution in distance color maps between the predicted skull meshes and the reconstructed skull meshes. The horizontal numbers indicate the tested subject identification.

Figure 6 shows the validation results between the predicted skull meshes and the CT-reconstructed skull meshes in shape and mesh differences. Overall, as shown in Figure 6a, the predicted skull meshes (represented in wire-frame rendering) are optimally fitted with the reconstructed skull meshes (represented in smooth rendering). Regarding the skull shape comparison, as shown in Figure 6b, most deviations are distributed on the back skull regions and small regions of interest (i.e., teeth, top nose). Large deviations are distributed on the back head regions of subject 1 due to the head deformation during the CT image acquisition. Regarding the skull mesh comparison, as shown in Figure 6c, we can also have good accuracies on the facial regions of the skull meshes. Deviations also focused on the back-skull and internal regions of the skull meshes.

**Figure 7:**
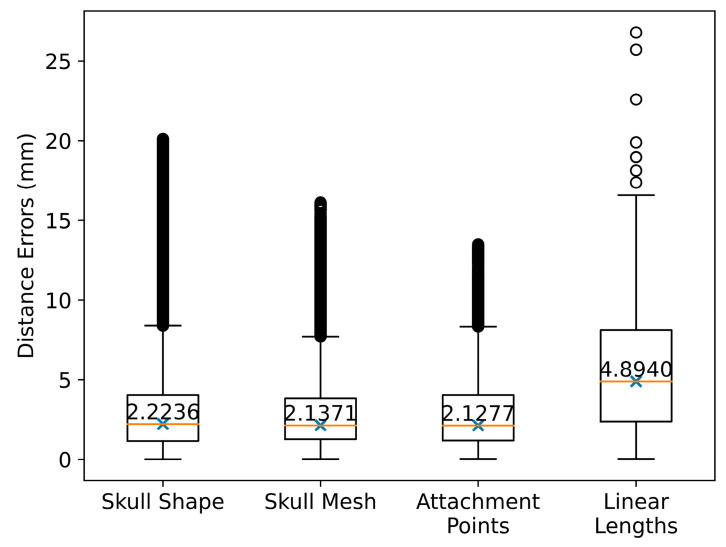
Validated deviations: generated vs. reconstructed skull shapes, generated vs. reconstructed skull meshes, generated vs. reconstructed skull meshes in muscle attachment point regions, and automatically generated vs. manually defined muscle lengths. Note that the circles represent the outliers. The “x”s and the numbers inside the boxplots indicate the mean values.

Figure 7 illustrates the deviations between the predicted skulls and the reconstructed skulls. In particular, mesh-to-mesh distances between the predicted skull shapes and the reconstructed skull shapes have a median of 2.2236 mm (Mean ± SD: 2.9917 ± 2.5117 mm). The median mesh-to-mesh distance between the predicted skull meshes and the reconstructed skull meshes is 2.1371 mm (Mean ± SD: 2.8694 ± 2.2194 mm). The median deviation in the muscle attachment points is 2.2177 mm (Mean ± SD: 2.9114 ± 2.2849 mm). The muscle length deviations have a median of 4.8940 mm (Mean ± SD: 6.1515 ± 5.1011 mm). This median deviation of the muscle lengths is within the experimental deviations of the muscle length (D ≈ 6 mm) as reported in the literature [65].

### 3.2. Muscle Lengths in Neutral Mimics

Table 1 shows the means and standard deviations of all muscle lengths of the 10,000 male and female subjects in this study. Overall, the mean muscle lengths of the male subjects are larger than those of the female subjects. The computed muscle lengths of the healthy subjects in our previous study are in the order of magnitude as those of the virtual subjects in this study. The average standard deviation of all muscle lengths is 2.7 mm, which agrees with the experimental perturbation values of the muscle insertion/attachment points (R ≈ 3 mm) as reported in the literature [65].

### 3.3. Static Muscle Analysis

Table 2 lists the standard strain values of the linear and circle muscles in smiling, kissing, and o-pronouncing mimics of all male and female subjects. Overall, the behaviors of the muscle strains can be used for describing and evaluating the quality of different facial mimics.

Regarding the smiling mimics, which the LZm, RZm, LZM, and RZM are mainly responsible for [53], the standard strain values (%) of the LZm, RZm, LZM, and RZM are −10.50 ± 0.48, −11.49 ± 0.60, −12.34 ± 0.50, and −12.78 ± 0.60, respectively, for males and −10.41 ± 0.47, −11.32 ± 0.57, −12.34 ± 0.52, and −12.74 ± 0.60, for females. Based on these strain values, the left and right Zm and ZM muscles are all shortened during the smiling mimics. This behavior is in agreement with the left and right Zm and ZM’s smiling behaviors reported in the literature [4,5]. Moreover, these strain values are relatively symmetrical between the left and right sides. These symmetrical and standard strain values could be used for evaluating the smiling mimics for healthy subjects, which usually have symmetrical and strong smiling mimics [4,5], and facial palsy patients, which usually have asymmetrical and weak smiling mimics [18]. For instance, in our previous study [36], for healthy subject 3, the strain values of the LZm, RZm, LZM, and RZM muscles were −9.93%, −9.93, −21.32%, and −19.72%, respectively. For patient subject 1, these values were −0.40%, −3.12%, −6.76%, and −9.53%, respectively. We can have a conclusion that the muscle strains of the healthy subject are stronger and more symmetrical than those of the patient subject when compared with the standard muscle strain values. Regarding the kissing mimics, for which the LZm, RZm, LZM, and RZM muscles are mainly responsible [53]. In the kissing mimic, these muscles must be elongated [4,5]. In this study, the standard strain values are 10.64% ± 0.48%, 11.69% ± 0.61%, 12.66% ± 0.50%, and 13.23% ± 0.59% for the LZm, RZm, LZM, and RZM muscle, respectively. Consequently, these values are all larger than zero and can show the elongating behavior during the kissing mimic. Moreover, we can see the symmetrical strain values of Zm and ZM muscles on the left and right sides. Consequently, these characteristics of the standard kissing strain values could also be used for evaluating the muscles in kissing mimics.

### 3.4. Dynamic Muscle Analysis

Besides the muscle evaluation in static mimics, this study also reported the dynamic muscle strain values supporting the muscle movement control diagnosis in smiling and kissing mimics. 

**Figure 8:**
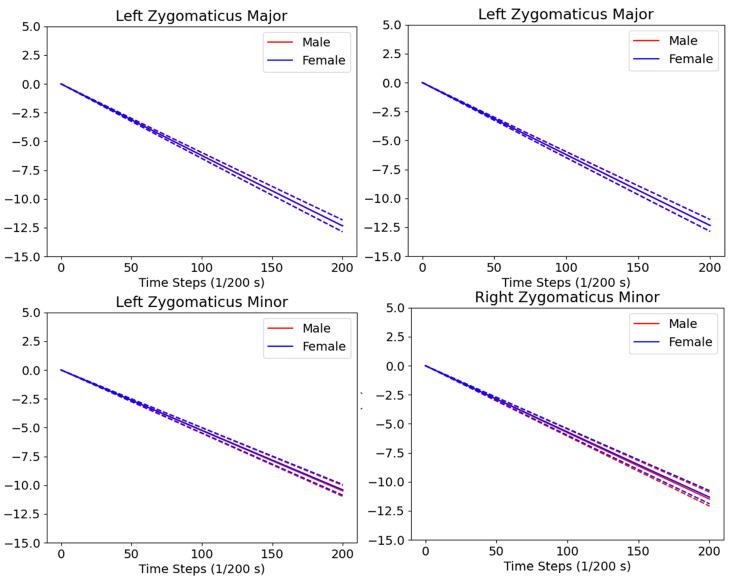
Dynamic strains of the left and right zygomaticus major and minor muscles in dynamical smiling mimics.

Figure 8 shows the dynamic behaviors of the LZm, RZm, LZM, and RZM muscles while performing the smiling mimics. Overall, these muscles are all shortening linearly when the mimic is performing from neutral to the max smiling range. This shortening behavior is suitable with the reported behaviors of the left and right ZM(m) muscles in smiling mimics [4,5,53]. The strain values in the left and right ZM(m) of the male and female subjects are relatively the same as each other during performing the smiling mimics. Moreover, the standard deviations of the muscle strains tend to increase when the mean shortening strain increase. Especially, the lengths of the left and right ZM muscles shorten faster than those of the left and right Zm muscles. For example, for the male subjects, at the time-step 0, the mean strains of the left and right ZM muscles and those of the left and right Zm muscles are all 0s. At the time-step 200, the strains of the left and right ZM muscles are −12.34% ± 0.50% and −12.78% ± 0.60%, which are all smaller than those of the left and right Zm muscles (−10.50 ± 0.48 and −11.49 ± 0.60, respectively). Additionally, from the time-step 0 to 200, the dynamic strains of the left and right ZM(m) muscles are relatively symmetrical to each other.

**Figure 9:**
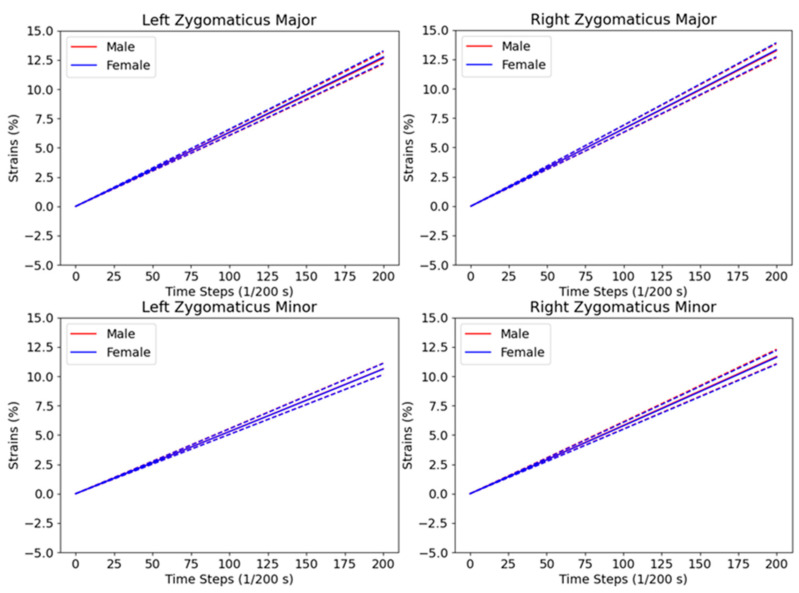
Dynamic strains of the left and right zygomaticus major and minor muscles in dynamical kissing mimics.

Figure 9 shows the dynamic behaviors of the LZm, RZm, LZM, and RZM muscles while performing the kissing mimics. As reported in the literature, during the kissing mimics, these muscles should be elongated proportionally to the kissing intensity [4,5,53]. This behavior is met with our computed strain values. In particular, the strains (%) of the left and right ZM muscles increase linearly from 0 s to 12.66 ± 0.50 and 13.23 ± 0.59, respectively, for males (12.75 ± 0.52 and 13.31 ± 0.60, respectively, for females) when the kissing mimics increase from the neutral to the max intensity. The left and right Zm muscles also increase linearly from 0 s to 10.64% ± 0.48% and 11.69% ± 0.61%, respectively, for males (10.62% ± 0.48% and 11.63% ± 0.59%, respectively, for females) during this kissing intensity range. It is important to note that the left and right ZM muscles elongate faster than the left and right Zm muscles during the kissing performing mimics, and the standard deviations of the elongation tend to be larger in higher strain values. Moreover, the left and right ZM(m) muscles also elongate symmetrically during the kissing.

Based on the above analyses of the LZm, RZm, LZM, and RZM muscles in dynamic smiling and kissing mimics, we can employ the computed muscle strains for scoring the movement of muscles responsible for specific dynamic mimics. Figure 8 and Figure 9 only show the analyzed results of the four muscles responsible for smiling and kissing mimics, but, in our dataset, we also reported strain values of the 37 muscles, as listed in Table 1, for these mimics.

## 4. Discussion

Facial paralysis grading is the first requirement for personalizing and enhancing facial mimic rehabilitation treatments [66]. Currently, non-clinical grading methods have been promisingly employed in this issue due to their stable and objective outcomes [17]. However, most studies just tried to analyze facial mimics based on their visual deformation on facial skin [20]. Facial mimics are the deformation results of muscle contractions on skin layers [3,4,5], so they should be directly analyzed and graded. Analyzing facial muscles during facial movements is relatively challenging because internal structures cannot be easily examined on living objects [36]. For example, surface scanning sensors (i.e., camera and time-of-flight sensors) cannot acquire internal structures [67,68,69]. Reconstruction of soft tissue from the MRI datasets is time-consuming and needs too much clinical expertise [4,5], and we cannot build face muscles from CT images [36]. Experimental processing on the decedent is limited and cannot acquire data on their facial mimics [38,39,40]. Recently, in our previous study, we proposed a novel method for analyzing facial muscles in real time based only on visual facial mimics supporting facial paralysis grading [36]. However, we lack the baseline datasets for automatically diagnosing the predicted muscles in static and dynamic facial mimics. Consequently, such standard parameters of facial muscles in different mimics are particularly necessary. Especially, in silico trials have been popularly applied for collecting large variations in datasets [41,42]. Consequently, in the present study, we developed a novel baseline muscle database using in silico trial approach to provide the first reference database for facial muscle evaluation.

More precisely, we first applied our head-to-skull and muscle prediction methods to a 3DMM head model [43], for providing its meshed skull and muscle networks throughout large shape- and expression parameter sets with acceptable accuracy for facial muscle analyses. The FLAME head model [43] was trained on a large database of 3800 scanned heads from the European CAESAR body scan database [51] and 3D face scans D3DFACS in various facial mimics [52]. Consequently, the FLAME model can be used for re-generating standard heads with mimics of the public. Statistical shape modeling was successfully employed to reduce the diversities of the training datasets and can deal with the lack of training data [70]. Therefore, with the small number of shape parameters, we could regenerate virtual head shapes, which represented the public head shapes. However, the model lacks internal structures. In this study, we first applied our novel head-to-skull prediction for inferring skull structures based on the head geometrical structures and predicting their muscle networks for analyzing muscle strains according to the FLAME head’s animations. After validating with 5 CT subjects, the muscle length deviations have a median of 4.8940 mm. These deviations are within the error range of the facial muscle lengths reported in the literature (D ≈ 6 mm). Moreover, the computed muscle length values in neutral mimics are compatible with the reported values in our previous study and the literature, as shown in Table 3. Particularly, in the literature, all experimental studies just computed the muscle lengths of a limited number of subjects (from 1 to 20). In our study, by using the SSM of the head, we could analyze the muscle lengths of a large number of subjects (males: 5000 and females: 5000). Consequently, our computed muscle lengths could be the reference values for muscle length diagnosing in neutral mimics. The computed muscle strains in smiling and kissing mimics are also well-matched with those computed using the accurate FE-based facial models and the Kinect-based head model. In particular, in smiling mimics of the FE-based model, the strain value of the left and right ZM muscles was reported as −6.82%. In the smiling mimic of the Kinect-based model, the strain values of the left and right ZM muscles of the healthy subject were −17.46% ± 3.87% and −14.43% ± 5.30%, respectively. In this study, as listed in Table 2, the strains of the left and right ZMs are −12.34% ± 0.50% and −12.78% ± 0.60%, respectively, for males (−12.34% ± 0.52% and −12.74% ± 0.60%, respectively, for females). In the kissing mimics, as reported in the FE-based models, the ZM strain values were 24% [4] and 22% [5] when the subject made the [o]-sound, which is relatively similar to the kissing mimic [53]. In the Kinect-based model, these strain values were 14.84% ± 2.56% for the left and right ZMs of the healthy subjects. In this study, the kissing strain values of the left and right ZMs are 12.66% ± 0.50% and 13.23% ± 0.59%, respectively, for males (12.75% ± 0.52% and 13.31% ± 0.60%, respectively, for females). With the acceptable accuracy of the muscle behavior analyses in this study, we first proposed to use the reported values of muscle lengths and strains for facial muscle diagnosis in facial paralysis grading. By using our previous patient-specific real-time head animation, head-to-skull prediction, and muscle network definition methods, the strain of each muscle could be computed in real-time. The static or dynamic muscle strains recorded during performing the smiling or kissing mimic will be compared with the standard static or dynamic muscle strain values of the smiling or kissing mimic for muscle diagnosis. Moreover, as listed in Table 2, we found that the standard deviations of the muscle strain in smiling and kissing mimics are relatively small among various subjects. This means that we can use the report values for evaluating the muscles of the large population during their mimic performance. Additionally, as shown in Figure 8 and Figure 9, standard deviations of the muscle strains tend to increase when increasing the intensity of smiling or kissing mimics. This information is important for evaluating muscle synkinesis while performing mimics [8].

In addition, we provided a large database supporting facial paralysis grading and facial muscle diagnosis. This database includes 10,000 FLAME-based head meshes and 10,000 skull meshes predicted from the head meshes. This dataset could be used for studying relations between the FLAME head shape parameters and the skull structures. Moreover, muscle lengths can be straightforwardly computed from the FLAME head and skull meshes by directly changing the expression parameters of the FLAME model. Muscle lengths can, therefore, be analyzed according to the FLAME expression parameters. We also provided muscle lengths of all muscles of all 10,000 analyzed subjects in neutral mimics. Muscle strains of all muscles in smiling and kissing mimics were also provided for all subjects. Means and standard deviations of muscle lengths, smiling strains, and kissing strains were also reported separately for all males and females. The database could be downloaded via the link [71].

This study also contains some drawbacks. We only analyzed and report muscle strain values on static and dynamic smiling and kissing mimics. However, the method can be applied to analyze the muscle in any mimics by controlling the expression and pose parameters of the FLAME model. Moreover, our muscle-based analyzing method has not supported mimics with mandible movements. In perspective, we will enhance the muscle-based facial analysis so that it can support the facial mimics with mandible movement by studying the relation between the mouth movements and the mandible motions. The enhanced method will be used for analyzing the muscle of all Action Units (AUs) in the Facial Action Coding System (FACS). We will also implement the method into our clinical decision-support system [72] for automatically detecting malfunctioning muscles while performing each AU of FACS. Based on the diagnosed results, we will propose suitable serious games for training the target muscle. It is also important to note that the statistical shape modeling methods were employed for building the FLAME and head-to-skull prediction models. In these statistical models, geometrical deformations of the meshes were computed as linear combinations of the principal components, so they cannot handle complex geometrical structures of the head and skull meshes. More advanced statistical shape modeling methods (e.g., Gaussian-based PCA [73]), geometric deep learning [74], and Generative Adversarial Networks (e.g., SP-GAN [75]) can be employed to solve these issues. In further works, we will implement the computed muscle strains as the baseline values for diagnosing facial muscle behaviors. In particular, in our previous study, we could compute patient-specific muscle strains in real-time. For diagnosing, clinicians will then ask the patient to perform smiling and kissing mimics. Strains of all muscles were computed in those mimics. The failed muscles are those having computed strain values different from the baseline strain values of these muscles. The facial mimic rehabilitation exercises will then be selected to train the failed muscles.

## 5. Conclusions

Facial paralysis grading is important for personalizing facial mimic rehabilitation treatments. Muscle-based facial grading method has recently been proposed in the literature, but there is still a lack of facial muscle baseline data to evaluate the patient states and guide as well as optimize the rehabilitation strategy. In this present study, we aimed to develop a novel baseline facial muscle database (static and dynamic behaviors) using the coupling between statistical shape modeling and in-silico trial approaches. We applied our original head-to-skull and muscle network prediction method to the FLAME model, which can be represented for the standard head shapes and facial mimics, for computing the standard muscle strains in both static and dynamic smiling and kissing facial mimics. In perspective, this data will be integrated into our available clinical decision support system for automatically detecting malfunctioning muscles and proposing patient-specific rehabilitation serious games.

## Figures and Tables

**Table 1:** Muscle lengths and perimeters of linear and circle muscles in neutral mimics of male and female subjects in this study and our previous study.

Left/Rights	Muscle Types	Muscle IDs *	$\mathrm{Action}\;\mathrm{Line}\;\mathrm{Lengths}\;\mathrm{of}\;\mathrm{Facial}\;\mathrm{Muscles}\;\mathrm{in}\;\mathrm{Neutral}\;\mathrm{Position}\;(l_{0})(\mathrm{Mean}\;\pm \;\mathrm{SD}\;**\;\mathrm{mm})$	
			Males	Females
Left	Procerus	LP	27.76 ± 2.41	27.56 ± 2.26
Right		RP	26.58 ± 2.33	26.63 ± 2.26
Left	Frontal Belly	LFB	33.06 ± 1.84	32.07 ± 1.77
Right		RFB	32.60 ± 1.78	31.74 ± 1.79
Left	Temporoparietalis	LT	30.69 ± 1.73	30.10 ± 1.70
Right		RT	22.89 ± 1.77	22.28 ± 1.79
Left	Corrugator Supperciliary	LCS	29.83 ± 1.64	29.55 ± 1.67
Right		RCS	28.45 ± 1.69	28.26 ± 1.84
Left	Nasalis	LNa	28.22 ± 1.94	27.56 ± 2.09
Right		RNa	29.05 ± 1.92	28.25 ± 2.12
Left	Depressor Septi Nasi	LDSN	17.12 ± 2.66	16.39 ± 2.31
Right		RDSN	13.29 ± 2.38	13.11 ± 2.35
Left	Zygomaticus Minor	LZm	59.21 ± 2.82	55.88 ± 2.74
Right		RZm	54.73 ± 2.89	52.25 ± 2.85
Left	Left Zygomaticus Major	LZM	67.29 ± 2.71	63.70 ± 2.62
Right		RZM	62.93 ± 2.71	59.81 ± 2.68
Left	Risorius	LR	36.84 ± 2.05	37.19 ± 1.98
Right		RR	36.04 ± 2.05	36.75 ± 1.88
Left	Depressor Anguli Oris	LDAO	31.19 ± 3.17	30.19 ± 2.17
Right		RDAO	32.39 ± 3.64	31.47 ± 2.65
Left	Mentalis	LMe	26.49 ± 3.23	26.76 ± 2.63
Right		RMe	29.07 ± 3.14	29.00 ± 2.60
Left	Levator Labii Superioris	LLLS	50.28 ± 3.01	47.05 ± 2.87
Right		RLLS	46.54 ± 2.75	44.01 ± 2.82
Left	Levator Labii Superioris Alaeque Nasi	LLLSAN	60.39 ± 2.83	57.16 ± 2.81
Right		RLLSAN	59.65 ± 2.74	56.63 ± 2.82
Left	Levator Anguli Oris	LLAO	38.14 ± 2.87	34.96 ± 2.82
Right		RLAO	34.49 ± 2.80	31.76 ± 2.76
Left	Depressor Labii Inferioris	LDLI	37.39 ± 2.20	37.33 ± 2.19
Right		RDLI	35.99 ± 2.65	35.52 ± 2.22
Left	Buccinator	LB	55.65 ± 3.03	53.54 ± 3.05
Right		RB	52.32 ± 3.09	50.55 ± 2.94
Left	Masseter	LMa	49.45 ± 2.38	47.02 ± 2.20
Right		RMa	52.14 ± 2.56	49.68 ± 2.23
Left	Orbicularis Oculi	LOO	153.60 ± 5.01	150.45 ± 5.25
Right		ROO	148.66 ± 4.70	144.12 ± 4.81
	Orbicularis Oris	OO	176.43 ± 7.94	165.76 ± 6.36

* ID: Identification; ** SD: Standard Deviation.

**Table 2:** Muscle strains of males and females subjects in static mimics: smiling and kissing.

Muscle IDs	Muscle Strains in Positions $\left(\frac{l-l_{0}}{l_{0}}\right)$ (Mean ± SD %)			
	Smiling		Kissing	
	Males	Females	Males	Females
LP	3.06 ± 0.34	3.00 ± 0.26	−3.05 ± 0.34	−3.00 ± 0.26
RP	3.39 ± 0.38	3.30 ± 0.30	−3.38 ± 0.38	−3.30 ± 0.30
LFB	2.88 ± 0.21	2.87 ± 0.21	−2.84 ± 0.21	−2.84 ± 0.21
RFB	2.52 ± 0.17	2.56 ± 0.16	−2.49 ± 0.17	−2.53 ± 0.16
LT	2.50 ± 0.20	2.46 ± 0.19	−2.47 ± 0.20	−2.44 ± 0.19
RT	3.05 ± 0.29	3.07 ± 0.27	−3.02 ± 0.28	−3.05 ± 0.26
LCS	−0.26 ± 0.30	−0.05 ± 0.26	0.35 ± 0.30	0.14 ± 0.26
RCS	−0.50 ± 0.44	−0.19 ± 0.38	0.63 ± 0.44	0.32 ± 0.38
LNa	−9.69 ± 1.03	−9.40 ± 0.95	10.44 ± 1.10	10.08 ± 1.01
RNa	−6.72 ± 0.77	−6.97 ± 0.72	7.74 ± 0.80	7.86 ± 0.78
LDSN	−22.40 ± 4.18	−22.76 ± 3.70	22.88 ± 4.49	23.12 ± 3.78
RDSN	−21.63 ± 3.86	−22.83 ± 3.85	25.41 ± 4.43	25.95 ± 4.37
LZm	−10.50 ± 0.48	−10.41 ± 0.47	10.64 ± 0.48	10.62 ± 0.48
RZm	−11.49 ± 0.60	−11.32 ± 0.57	11.69 ± 0.61	11.63 ± 0.59
LZM	−12.34 ± 0.50	−12.34 ± 0.52	12.66 ± 0.50	12.75 ± 0.52
RZM	−12.78 ± 0.60	−12.74 ± 0.60	13.23 ± 0.59	13.31 ± 0.60
LR	−12.17 ± 1.72	−12.92 ± 1.63	13.88 ± 1.64	14.55 ± 1.63
RR	−10.57 ± 2.02	−11.49 ± 1.72	12.76 ± 2.00	13.55 ± 1.76
LDAO	5.23 ± 2.53	4.33 ± 3.09	1.32 ± 2.78	3.47 ± 2.98
RDAO	9.91 ± 3.01	9.92 ± 3.34	−5.18 ± 2.23	−3.95 ± 2.61
LMe	−0.14 ± 1.46	−0.79 ± 1.87	2.82 ± 2.04	4.03 ± 2.29
RMe	1.31 ± 1.14	0.90 ± 1.59	0.88 ± 1.12	1.77 ± 1.46
LLLS	−11.76 ± 0.77	−11.57 ± 0.75	12.13 ± 0.77	12.08 ± 0.75
RLLS	−12.24 ± 0.95	−11.96 ± 0.90	12.87 ± 0.96	12.74 ± 0.91
LLLSAN	−8.05 ± 0.45	−7.77 ± 0.51	8.65 ± 0.46	8.48 ± 0.50
RLLSAN	−7.44 ± 0.47	−7.21 ± 0.51	8.17 ± 0.47	8.03 ± 0.49
LLAO	−22.01 ± 1.86	−22.67 ± 2.01	22.82 ± 1.86	23.66 ± 2.05
RLAO	−19.84 ± 2.20	−20.62 ± 2.19	22.23 ± 2.38	23.14 ± 2.34
LDLI	−7.46 ± 1.30	−8.20 ± 1.27	8.25 ± 1.30	8.98 ± 1.29
RDLI	−6.55 ± 1.68	−7.82 ± 1.57	7.76 ± 1.77	9.04 ± 1.67
LB	−12.89 ± 0.79	−13.46 ± 0.89	13.57 ± 0.82	14.10 ± 0.95
RB	−12.21 ± 0.78	−12.77 ± 0.83	13.14 ± 0.84	13.66 ± 0.91
LMa	0.22 ± 0.49	−1.16 ± 0.58	0.02 ± 0.45	0.86 ± 0.54
RMa	−0.65 ± 0.48	−0.25 ± 0.57	0.74 ± 0.46	0.14 ± 0.57
LOO	−2.14 ± 0.10	−2.16 ± 0.11	2.27 ± 0.11	2.29 ± 0.11
ROO	−2.31 ± 0.10	−2.39 ± 0.11	2.46 ± 0.10	2.54 ± 0.12
OO	13.18 ± 0.68	14.46 ± 0.66	−11.31 ± 0.64	−12.44 ± 0.61

**Table 3:** Comparison between the muscle lengths computed in this study and our previous study and the literature.

Muscle IDs	Lengths of Facial Muscles in Neutral Mimics Reported in the Literature													
	This Study *				Nguyen et al., 2021 [36]		Freilinger et al., 1987 [38]		Happak et al., 1997 [39]		Bernington et al., 1999 [40]		Fan et al., 2017 [4]	Dao et al., 2018 [5]
	Subjects: 5000 M, 5000 F Ages: 29–49 Years Status: In Silico				Subjects: 2 M, 3 F Ages: 29–49 Status: 3 H, 2 P Weight: 52–71 Kg Height: 1.65–1.77 m BMI: 18–26 kg/m^2^		Subjects: 20 Ages: 62–94 Status: Cadavers		Subject: 11 Ages: 53–73 Years Status: Cadavers		Subjects: 4 M, 6 F Ages: 15–31 Status: Patients		Subject: 1 F Ages: 24 Status: Healthy Height: 1.5 m Weight: 57 kg	
	Males		Females											
	Mean	SD	Mean	SD	Mean	SD	Mean	SD	Mean	SD	Mean	SD	Value	Value
LZm	59.21	2.82	55.88	2.74	51.05	3.82	-	-	51.8	7.4	-	-	-	-
RZm	54.73	2.89	52.25	2.85	53.90	2.05	-	-	51.8	7.4	-	-	-	-
LZM	67.29	2.71	63.70	2.62	58.45	3.85	M: 0.67 F: 69.50	6.32 6.58	65.6	3.8	-	-	43.65	52
RZM	62.93	2.71	59.81	2.68	61.23	3.05	M: 0.67 F: 69.50	6.32 6.58	65.6	3.8	-	-	43.65	52
LDAO	31.19	3.17	30.19	2.17	36.69	3.23	M: 37.83 F: 38.33	4.38 8.02	48	5.1	-	-	-	-
RDAO	32.39	3.64	31.47	2.65	31.86	3.35	M: 37.83 F: 38.33	4.38 8.02	48	5.1	-	-	-	-
LLLS	50.28	3.01	47.05	2.87	46.26	3.00	M: 33.67 F: 35.50	4.13 6.69	47	7.5	-	-	29.3	-
RLLS	46.54	2.75	44.01	2.82	48.59	2.14	M: 33.67 F: 35.50	4.13 6.69	47	7.5	-	-	29.3	-
LLLSAN	60.39	2.83	57.16	2.81	58.06	3.65	-	-	61.6	7.6	-	-	-	-
RLLSAN	59.65	2.74	56.63	2.82	59.46	2.81	-	-	61.6	7.6	-	-	-	-
LLAO	38.14	2.87	34.96	2.82	34.30	2.53	-	-	42	2.5	-	-	27.4	-
RLAO	34.49	2.80	31.76	2.76	35.51	2.30	-	-	42	2.5	-	-	27.4	-
LDLI	37.39	2.20	37.33	2.19	36.73	4.39	-	-	29	4.9	-	-	-	-
RDLI	35.99	2.65	35.52	2.22	37.01	4.16	-	-	29	4.9	-	-	-	-
LB	55.65	3.03	53.54	3.05	56.35	3.35	-	-	56	7.4	-	-	-	-
RB	52.32	3.09	50.55	2.94	55.18	2.01	-	-	56	7.4	-	-	-	-
LMa	49.45	2.38	47.02	2.20	44.93	2.35	-	-	-	-	M: 45.9 F: 39.1	5.8 8.2	-	-
RMa	52.14	2.56	49.68	2.23	45.03	2.57	-	-	-	-	M: 45.9 F: 39.1	5.8 8.2	-	-
VLOO	51.81	1.92	50.46	2.03	40.70	2.99	-	-	60	9.6	-		-	-
VROO	46.72	1.63	45.29	1.93	41.62	2.13	-	-	60	9.6	-		-	-
HLOO	36.47	1.73	35.68	1.67	56.53	3.23	-	-	65	5.6	-		-	-
HROO	36.48	1.75	35.82	1.78	56.92	2.85	-	-	65	5.6	-		-	-

* M: Male; F: Female; Ages: Min–Max (Years Old).

## Data Availability

Data is available upon request.
